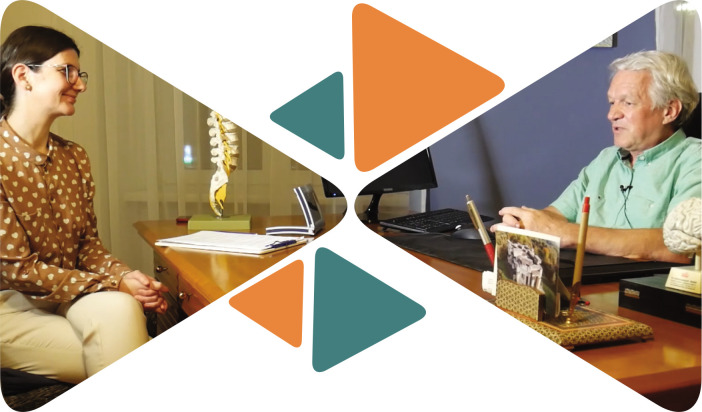# Christian Matula – Adapted interviews from Neurotrauma Treatment Simulation Center (NTSC) – VIENNA, 2022

**DOI:** 10.25122/jml-2022-1019

**Published:** 2022-07

**Authors:** Alexandra-Mihaela Gherman, Andreea Doria Constantinescu, Andreea Strilciuc

**Affiliations:** 1. RoNeuro Institute for Neurological Research and Diagnostic, Cluj-Napoca, Romania

**Interviewee: Christian MATULA**

**Interviewer: Doria CONSTANTINESCU**

**D.C**.: Hello, Professor Matula and thank you for being such a great host for us here, in Vienna, for the Neurotrauma Treatment Simulation Center (NTSC). If you could tell us a few words about yourself, including your area of research and practice.

**C.M**: First of all, thank you very much for coming and joining us, it's a pleasure having you all here. As you know, I am one of the grey eminences here, in Vienna, so I have been working here for a very long time, myself, alongside the fact that I am the wise chair here, from the whole Department, I am the director of the Skull Base Unit and the director for the Neurotrauma Center here, in Vienna, and I am very proud of that, very proud of having a great team working with me. Vienna is not only a great city, but it's also in the centre of Europe – we are working in the General Hospital in Vienna which is one of the biggest hospitals in Europe. We have around 2,500 beds here, we have around 14,000 people working in that hospital, including around 2,500 doctors. So, it's really a *city in the city*, and we are very proud of our institutions. We are treating patients not only from Austria but from all over the world. That makes us very proud and our main philosophy on what we are doing here is to have a strict focus on interdisciplinary work. So, we are a multidisciplinary institution and I am a neurosurgeon from my heart. I always used to say 'I'm a bloody surgeon, I'm a simple medical handworker'. And I'm very proud of it.

**D.C**.: What was your motivation for taking such a good lead in this coordinating team for the Program?

**C.M**.: There is one simple answer for that **–** because I truly believe in that. I truly believe in that [the Program] because this is the way into a great future. I think this is the way for the next steps, especially for the young generation. Together we are strong for our patients. And if we work together, then this is the best benefit our patients can have and definitely, that will improve outcomes.

**D.C**.: Could you describe how your experience shaped your opinion regarding the treatment of neurotrauma?

**C.M**.: First of all, experience is something that takes a while. Experience you can't get with a click, in a minute. The shapes, especially in terms of neurotrauma, are those unbelievable varieties of cases we are confronted with.

So, neurotrauma is something we are confronted with day by day. Those things can happen to anyone of us, at anytime, in any place. And dealing with that type of patient you have to be flexible, you have to have great empathy towards what's going on and actually, you have to prepare to do something that people may say you never can do. You can.

**D.C**.: Are you familiar with any similar programs running at the moment? Like the one we are in right now?

**C.M**.: The NTSC VIENNA project is worldwide unique. The Neurotrauma Treatment Center Vienna is something we created based on a lot of conversations we had before, it's based on competence and driven by excellence. It means that we have been lucky to bring the right people together and so the Neurotrauma Center, the Neurotrauma Treatment Simulation Center, to be correct, is something worldwide unique and we are very proud of that.

**Figure:**
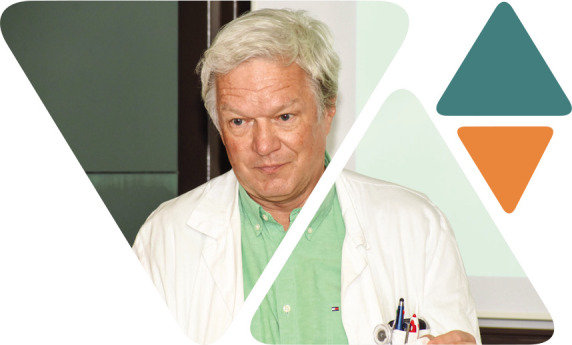


**D.C**.: If you had to identify the main challenges in the approach of TBI in the current times, which three (3) of these would be on top?

**C.M**.: That has changed over the years. And, if you listened to my talks today, the expectations of the patient from the past, have simply switched [...]. The expectations of the patients of the present are like, as I call it – “the McDonald's surgery”. It means they come in to be treated immediately and leave the hospital at once. Sometimes even without paying. And the expectations of the patients of the future will be optimized. They will like to come to the hospital to be treated immediately and to be much better afterwards than before. They like to be optimised. And this is one of the major tasks for the future because the patients' expectations are very high. In terms of neurotrauma, in the future, we definitely should focus on motor recovery and depression. More than three-quarters of the patients will get depression after trauma. This is an enormously high number, this is a big group of people, so we will have to work on that. And motor function recovery is something that people really need in their daily life. So, these are the two focuses which we have to take seriously in our mind.

**D.C**.: Regarding the treatment and neurorehabilitation process for patients suffering from neurotrauma, which organisational issues do you consider to be most pressing?

**C.M**.: That sounds like such an easy question with so many difficult answers because the answers go in many directions. [...] I am a neurosurgeon, doing neurosurgery work **–** this is no longer enough. This is just one part of the story. If we look at the whole workflow of neurotrauma, there is the beginning of trauma, the first response, the first line rescue chain, the transportation to the centres, treatment within the centres, no matter if it's just consultative or surgically, the aftercare, follow-up, rehabilitation and reintegration in the patients' normal life and work. If just one part of that chain is failing, then all the work fails. So, a chain is as strong as its weakest link. [...] If we really like to cover the whole neurotrauma portfolio, then we have to look in all that stuff. All that includes, of course, rehabilitation, which is a very important part of the program. But it includes also prevention. Don't forget prevention. Because it shouldn't come to that sometimes often terrible accidents. A good example is wearing helmets. That has changed the landscape, the neurotrauma landscape.

**D.C**.: From your point of view, which are the main limitations in the approach to cognitive, behavioral, or depressive disorders resulting from neurotrauma?

**C.M**.: This is a good question and not easy to answer. But I will try. [...] The limitations are, in terms of severe trauma, the morphological damage. Unfortunately, if big parts of the brain are damaged, this is a limitation. Another limitation is the inadequate aftercare and follow-up situations. I mean, the treatment for trauma doesn't stop by removing an epidural. It goes much more after that. So, this is what I mentioned before. Once again, you have to have an eye on the whole chain to understand what is going on. And once again, a chain is as strong as its weakest link. And you have to take care that that chain has really strong links.

**Figure:**
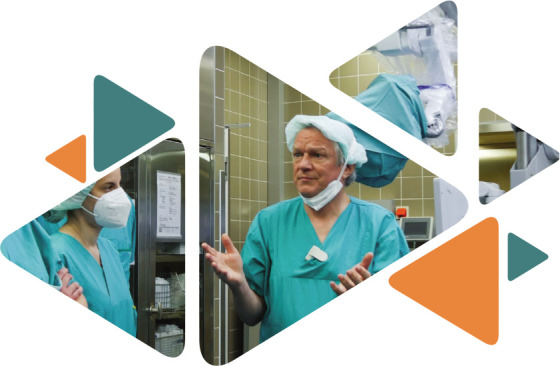


**Figure:**
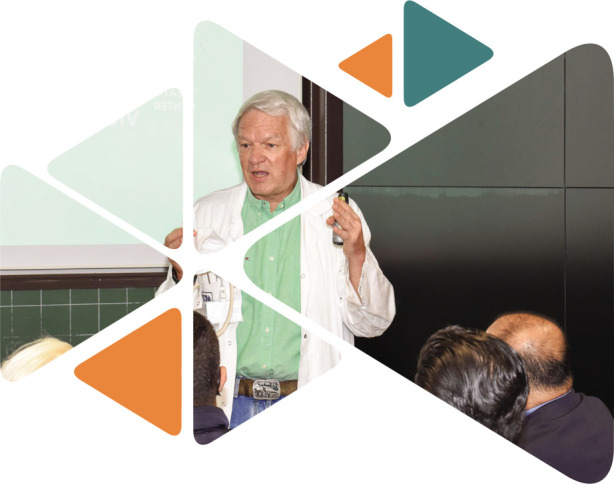


**D.C**.: Could you describe one of the most complex or intriguing cases of neurotrauma in your career so far? [...] Out of the many, of course. If there is one that stands out from the rest.

**C.M**.: I will honestly answer your question. As you know, we, as neurosurgeons, are confronted mostly with the real severe parts of neurotrauma. We know that about 90% of trauma are mild to moderate traumas [...] I remember a case I had, it was a young woman about 30–35 years of age, who was picking up her kids from the kindergarten, driving her car and she stopped at the traffic light and, besides her, her ex-husband stopped and shot her with a pump gun. As she was brought to our institution, more dead than alive, and I had a big fight with the chief of our Trauma Department because I said 'This is a young woman and if she has the minimum chance, we have to give her that chance'. And I remember very good [...] all the posterior fosulla was blown away and I operated on her for about 20 hours, so I removed, I counted, I removed 62 bullets out of her head and her upper cervical spine. And we had to reconstruct everything from the bone, and I have pictures of that, where I have the last bullet directly at the brain stem. And at that moment, I was also not sure how it will end up. Today, she has a normal life. It's not only that she is alive, it's not only that she is a wonderful mother bringing up her kids, she is also very successful in her job, but she's also a wife without any deficits and that makes me really proud. And this is also one of my simple philosophies: Never give up!

**D.C**.: This is so interesting! The next questions will be very boring after that. Online communities in the medical field are becoming more and more visible. Considering this, what do you think of the role of online communities in medical practice? [...]

**C.M**.: Because we were confronted with the pandemic, at the beginning of the pandemic, I think this was a good thing to do. It brought the people together although they hadn't had the chance. Now, I have to say that most people are sick because of that. If I look at the participants, they had enough of that. They prefer real face-to-face meetings. What they want are not the big meetings, they like the smaller ones, they like the direct communication, they like the direct conversation, so to answer your question, I think, in principle, it is a very good thing. In principle, it goes very quickly, this is a new style but, at the end of the day, it's not as beneficial and not as efficient as a really good face-to-face meeting.

**D.C**.: From your perspective, which are the obstacles that would affect a proper long-term follow-up of patients?

**C.M**.: [...] Unfortunately, the success of medical research in neurotrauma was not really that high in the last decades. Now we are happy to have good working substances which are influencing the long-term outcome and I think this is very good hope for the future. So, there's a light at the horizon coming up. And I'm just following that light. I think all the things we have in addition to our surgical work, to our initial work in neurotrauma which goes far beyond that, are very helpful. The add-on therapy and the medications for the long run are very useful and very beneficial for the patients.

**D.C**.: Our last question is related to COVID-19 and we want to ask, how do you consider it impacted the management of neurotrauma?

**C.M**.: Yes, probably my answer will surprise you. Because my feeling, working in one of the biggest hospitals, involved in all that stuff, is that it didn't have that big effect. Or that big influence.

Because trauma happens with or without COVID-19.

So, if you ask me, is the number of cases decreasing because of COVID? I don't have the feeling. Probably the landscape is a little different. In Austria, people are crazy about riding their motorcycles and riding their mountain bikes and going skiing, in particular skiing.

During the pandemic, that has changed, [...] the problems were in the family, internally [...] Unfortunately, the suicide rate became higher. So, eventually, the landscape has changed a little bit, but not in the number.

**D.C**.: Thank you very much!

**C.M**.: It's my pleasure! [...]

Watch the extended interview on the AMN Website: https://brain-amn.org/ntsc-interviews-series-christian-matula-meduni-wien-austria/

**Figure:**